# An exome sequencing pipeline for identifying and genotyping common CNVs associated with disease with application to psoriasis

**DOI:** 10.1093/bioinformatics/bts379

**Published:** 2012-09-03

**Authors:** Lachlan J.M. Coin, Dandan Cao, Jingjing Ren, Xianbo Zuo, Liangdan Sun, Sen Yang, Xuejun Zhang, Yong Cui, Yingrui Li, Xin Jin, Jun Wang

**Affiliations:** ^1^BGI-Shenzhen, Shenzhen, 518083, China; ^2^Department of Genomics of Common Disease, Imperial College London, London W12 0NN, UK; ^3^Institute of Dermatology and Department of Dermatology, No.1 Hospital, Anhui Medical University, Hefei, Anhui, 230032, China; ^4^State Key Laboratory Incubation Base of Dermatology, Ministry of National Science and Technology, Hefei, Anhui, China; ^5^School of Bioscience and Biotechnology, South China University of Technology, Guangzhou 510641, China

## Abstract

**Motivation:** Despite the prevalence of copy number variation (CNV) in the human genome, only a handful of confirmed associations have been reported between common CNVs and complex disease. This may be partially attributed to the difficulty in accurately genotyping CNVs in large cohorts using array-based technologies. Exome sequencing is now widely being applied to case–control cohorts and presents an exciting opportunity to look for common CNVs associated with disease.

**Results:** We developed ExoCNVTest: an exome sequencing analysis pipeline to identify disease-associated CNVs and to generate absolute copy number genotypes at putatively associated loci. Our method re-discovered the *LCE3B_LCE3C* CNV association with psoriasis (*P*-value = 5 × 10*e*−6) while controlling inflation of test statistics (λ *<* 1). ExoCNVTest-derived absolute CNV genotypes were 97.4% concordant with PCR-derived genotypes at this locus.

**Availability and implementation:** ExoCNVTest has been implemented in Java and R and is freely available from www1.imperial.ac.uk/medicine/people/l.coin/.

**Contact:** wangj@genomics.org.cn or Lachlan.J.M.Coin@genomics.org.cn

## 1 INTRODUCTION

Common copy number variation (CNV) in the human genome has been extensively catalogued using both array comparative hybridization (aCGH) as well as more recently with next-generation whole genome sequencing (Conrad *et al.*, 2010; Mills *et al.*, 2011; Redon *et al.*, 2006). Studies have demonstrated that common CNV is prevalent in genic as well as non-genic regions of the genome and accounts for ~18% of detected genetic variation in gene expression (Stranger *et al.*, 2007). While common copy number variants associated with complex disease have been identified (Aitman *et al.*, 2006; de Cid *et al.*, 2009; Diskin *et al.*, 2009; McCarroll *et al.*, 2008), they are vastly fewer than reported single-nucleotide polymorphism (SNP) associations. In a landmark study, the Wellcome Trust Case Control Consortium identified the majority of common CNV with allele frequency *>*5% and genotyped this variation in seven complex disease cohorts and two control cohorts using micro-array-based comparative genomic hybridization (aCGH). However, analysis of these data revealed only three previously identified copy number associations (Craddock *et al.*, 2010). This study also comprehensively identified many sources of bias which continue to plague CNV association studies, including batch effects, systematic differences between DNA extracted from cell lines versus whole blood, homology, particularly to the MHC, as well as difficulty in accurately determining CNV genotypes. The authors of this study concluded that the subset of CNVs that are genotypeable on CGH arrays—mostly simple non-recurrent deletions—do not explain a large proportion of phenotypic variation. However, there remains the possibility of extensive disease association with more complex CNV, as well as common CNV with small to moderate effect sizes.

In contrast to most complex disease, the role of common CNV in the pathogenesis of psoriasis has been robustly demonstrated and replicated across multiple populations worldwide (de Cid *et al.*, 2009; Hollox *et al.*, 2008; Huffmeier *et al.*, 2010; Li *et al.*, 2011; Riveira-Munoz *et al.*, 2011; Wiwanitkit, 2010; Xu *et al.*, 2011). In particular, association between psoriasis and a deletion spanning the ‘late cornified envelope’ genes *LCE3B and LCE3C* was originally shown in European populations (de Cid *et al.*, 2009) and was subsequently replicated in a Chinese cohort (Xu *et al.*, 2011). This CNV is a simple 32.2-kb deletion, with allele frequency of ~60%, for which both heterozygote and homozygote deletions are found in the population. The *LCE3B_LCE3C* deletion has an odds ratio of ~1.38 for psoriasis.

Exome capture sequencing is now widely being applied to both Mendelian and complex disease cohorts (Ng *et al.*, 2010), primarily to find rare associations with disease which have not been identified by extensive GWAS (O'Roak *et al.*, 2011), as well as to refine association signals already reported (Bonnefond *et al.*, 2012; Palmer *et al.*, 2011; Rivas *et al.*, 2011). Exome sequencing uses hybridization technology to first capture the majority of exons, and then subsequently applies massively parallel sequencing to these captured fragments. As the sequencing step is a stochastic process, the number of times a sequence is read should be reflective of its relative copy number in the original sample. However, because of the hybridization step, many of the biases present in array-based studies, such as batch effects and, effects from obtaining DNA from different sources are still present in these data. Nevertheless, the utility of exome sequence data to examine CNV in matched tumour/healthy samples has already been demonstrated (Sathirapongsasuti *et al.*, 2011).

Exome capture may also be a promising approach for finding common copy number association with disease. However, the ability to accurately call absolute copy number genotypes from these data has yet to be demonstrated. Moreover, there has not yet been any demonstration of the ability to re-discover known common CNV associations from these data. In this article, we develop an approach to find common CNV associated with complex disease. We apply this approach to exome sequence data obtained on a psoriasis case–control cohort. We demonstrate that we are able to overcome inflation of test-statistics while identifying the known *LCE3B_LCE3C* deletion association with *P*-value of 5 × 10*e*−6 in a case–control cohort of 1500 samples. Moreover, we show that we can use exome sequence data to generate highly accurate absolute CNV genotypes at this locus.

## 2 METHODS

### 2.1 Exon capture and sequencing

Genomic DNA of each individual was hybridized with NimbleGen 2.1 M-probe sequence capture array to enrich the exonic DNA in each library. The array captures 18 654 of the 20 091 genes deposited in Consensus Coding Sequence Region database.

DNA was randomly fragmented by Covaris to an average size of 200–300 bp, and adaptors were ligated to both ends of the resulting fragments. The adaptor-ligated DNA products were amplified with different index primers. The same amount of PCR product from pairs of samples was pooled then hybridized to each capture array following NimbleGen's protocol, after which the exome-enriched DNA fragments were eluted from the array and amplified by ligation-mediated PCR, and non-hybridized fragments were then washed out.

Sequencing was carried out on the Illumina HiSeq 2000 platform for each captured library independently to ensure each sample had at least ~15-fold coverage. Raw image files were processed by Illumina Pipeline (version 1.3.4) for base-calling with default parameters and the sequences of each individual were generated as 90-bp reads.

SOAPaligner (v2.21) (Li *et al.*, 2008) was used to align the sequencing reads to the NCBI human genome reference assembly (build 36.3) with a maximum of three mismatches and the parameters set as ‘-a -b -D -o -u -r 1 -s 40 -l 35’.

### 2.2 Read depth normalization

Within each capture region i, and for each individual *k* = 1 … N, we record the total aligned read depth divided by the average read depth for that sample, which we label *r*_ijk_, for each base pair *j* = 1 … M. We then calculate the first ‘local’ principal component FPC_i,k_ for each matrix *R_i_* = {*r*_ijk_}, using an iterative scaling algorithm outlined below. This algorithm has a very low memory footprint and is highly computationally efficient. We then calculate the first 50 ‘global’principal components of the matrix comprised of all of the local first principal components {FPC_i,k_}, which we label GPC_1_ … GPC_50_. This calculation is carried out using the singular value decomposition, which is not as fast or memory efficient as the iterative scaling algorithm, but is more accurate for calculating lower order PCs. These global PCs are considered to capture biases which might lead to artefactual CNV association. We then calculate modified local PCs by projecting out the first *H* of these global PCs, for fixed *H*:
(1)
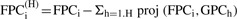

where
(2)
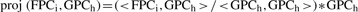

is the projection of the FPC_i_ onto the *h*^th^ global PC, using *< >* to denote the inner product in a *N* dimensional vector space.

### 2.3 Iterative scaling algorithm to calculate FPC

We have implemented an algorithm described in (Roweis, 1998) for computationally efficient calculation of the first principal component (FPC). We first centralize the matrix *R*_i_ so that each column has zero mean. We first FPC_i_ to be a vector of length *N* with entries randomly chosen from the interval (0,1). We then iteratively update FPC*_i_* via the following equations:
(3)
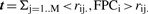

(4)
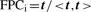

until convergence of FPC_i_. This algorithm avoids the computationally expensive calculation of the covariance matrix R*R^T^. Moreover, it has a very low memory footprint as the calculation in Equation (3) can be calculated via streaming one column of the depth matrix at a time. This algorithm accurately calculates the FPC but becomes progressively less accurate for lower order PCs.

### 2.4 Association with disease status

For a fixed level of adjustment, *H*, we then carry out association of FPC_i_^(H)^ over all regions *i*, with the case–control phenotype. The association is modelled with logistic regression, with *P*-values calculated using the GLM package in *R*. We plot quantile–quantile plots for different values of *H* and also calculate the genomic control inflation factor λ in order to inspect for inflation of test statistics.

### 2.5 Calculating absolute copy number genotypes

For a given putatively association region *i*, and for each sample, *k*, we calculate {*ra*_ijk_} as the average depth in non-overlapping 100-bp windows j divided by the genome-wide average depth. We then project out the first *H* global PCs to calculate
(5)
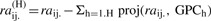


We then apply the population-haplotype clustering model of cnvHap (Coin *et al.*, 2010) to these data in order to jointly segment each sample into different CNV states. This is achieved by using a different emission distribution per hidden copy number genotype CN = 0 … 4 per 100-bp window to model the normalized read depth. Spatial modelling of the CNV is achieved via a HMM as previously described. CNVhap returns the CNV genotypes for each sample, as well as an estimate of the certainty of these genotypes.

## 3 RESULTS

**Fig. 1.**
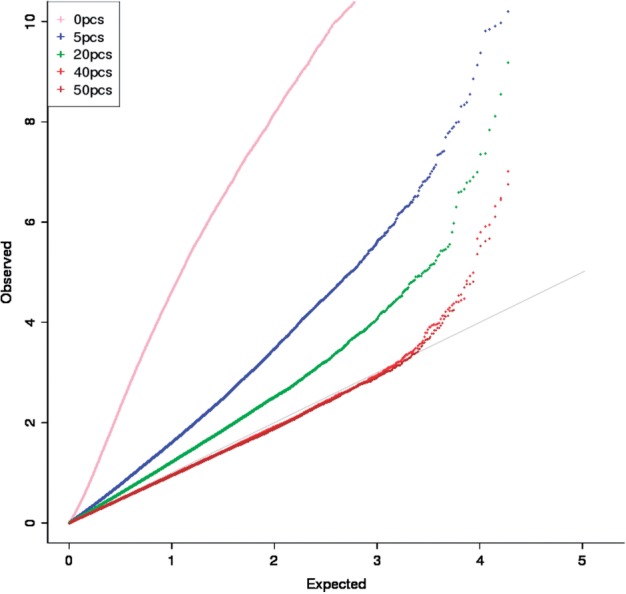
Quantile–quantile plot of association statistics. Observed versus expected –log10(p) test-statistic quantiles. Grey line indicates equality of observed and expected under the null hypothesis of no association. Pink, blue, green, red and maroon lines indicate observed quantiles after correcting for 0, 5, 20, 40 and 50 GPCs, respectively

We applied ExoCNVTest to exome sequence data collected on 800 controls and 700 psoriasis cases. Association of the uncorrected FPC was highly inflated with inflation factor (λ) of 8.5 (Fig. 1 and Table 1). In this analysis the known association *LCE3B_LCE3C* was ranked 3750 out of 105 088 regions. Correction for 5 and 20 GPCs progressively reduced inflation of test statistics and also increased the rank of *LCE3B_LCE3C*. However, correction for 40 GPCs was required to entirely remove inflation of test statistics and reduce λ *<* 1. In this analysis the *LCE3B_LCE3C* cluster was ranked 25th, with *P*-value of 7.2*e*−5.

**Table 1.** Association of FPC with psoriasis

GPCS	λ	*P*-value		Rank	
		LCE3B	LCE3C	LCE3B	LCE3C
0	8.51	1.3*e*−6	4.5*e*−7	5048	3750
5	1.88	1.3*e*−5	9.8*e*−6	238	204
20	1.28	2.0*e*−5	4.4*e*−5	56	82
40	0.92	7.2*e*−5	1.1*e*−4	25	33
50	0.91	1.4*e*−4	2.0*e*−4	31	35

Association *P*-value with case–control status of FPC after adjustment for 0, 5, 20, 40 and 50 GPCs. *P*-value rank out of 105 088 regions. Inflation factor λ calculated from median χ^2^ test statistic over all regions.

**Fig. 2.**
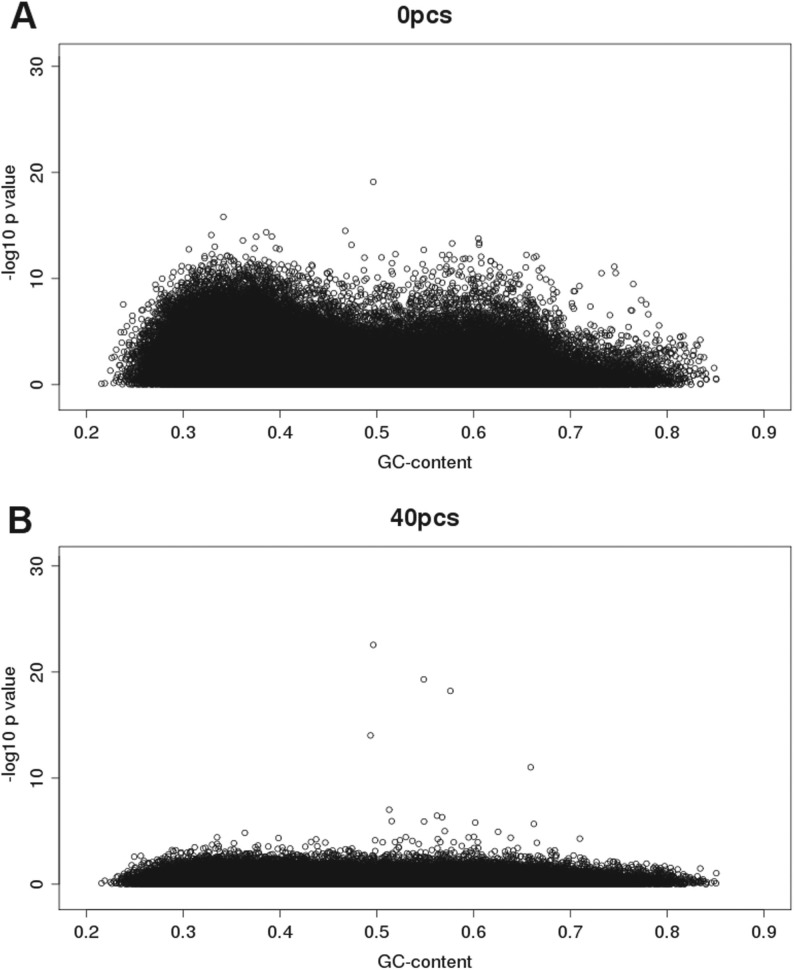
Significance versus GC-content before and after correction for 40 GPCS. Values for each of 105 088 regions indicating GC content and significance (−log 10 *P*-value). (**A**) without correction, (**B**) correcting for 40 GPCs

In order to investigate whether our approach was accurately capturing the underlying CNV present in *LCE3B_LCE3C*, we investigated the correlation between the adjusted FPC in these capture regions and gold standard copy number genotypes obtained from PCR in these samples. We observed increasing correlation between the adjusted FPC and the gold-standard PCR genotypes up to 20 GPC, but not beyond (Table 2). This demonstrates that adjusting for GPCs has two effects—the first being to reduce spurious signals and the second being to increase the correlation between real and detected signals at genuine associated loci, although the optimal number of GPC correction may be different for these two goals. We also observed substantial GC biases in uncorrected association statistics, with an over-representation of significantly associated signals at ~33% and 60% GC content (Fig. 2A). Correcting for 40 GPCs removed this bias (Fig. 2B).

**Table 2.** Correlation of FPC with PCR genotypes

#GPCS	Correlation with PCR	
	LCE3B	LCE3C
0	0.857	0.884
5	0.859	0.885
20	0.894	0.912
40	0.892	0.908
50	0.888	0.901

**Fig. 3.**
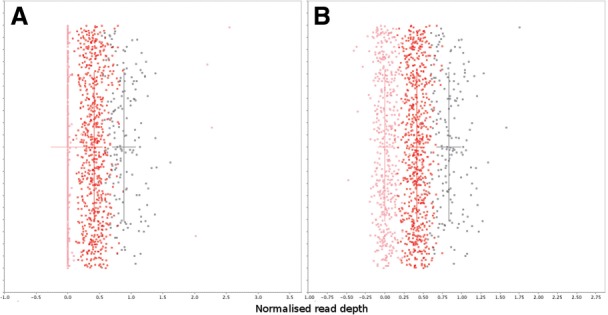
Normalized read depth per sample. Each dot indicates the average read-depth within the 100-bp window centred at 150,839,968bp on chromosome 1 divided by the average depth genome-wide for a given sample. Values on the *Y* -axis are randomly assigned between 0 and 1 to separate the samples. Dots are coloured by the CNV genotype assigned by cnvHap: pink = homozygous deletion; red = heterozygous deletion; black = normal. Crosses indicate mean and variance of clusters after 20 iterations of expectation maximization. Initial cluster positions were 0, 0.5, 1.0 for 0, 1, 2 copy number. (**A**) No correction for genome-wide PCs; (**B**) correction for 40 genome-wide PCs

Finally, we attempted to obtain absolute copy number genotypes at the *LCE3B_LCE3C* loci. We did this by averaging the per-sample read depth in 100-bp windows divided by average genome wide read depth and then projecting out the first 40 global PCs. These normalized data were presented to cnvHap, with initial copy number cluster positions at 0, 0.5, 1, 1.5, 2 normalized read depth for 0, 1, 2, 3, 4 copy numbers, respectively. cnvHap updates the positions of these cluster positions within a generalized expectation maximization framework and then reports the per-sample copy number genotypes (Fig. 3). The reported CNV genotypes were found to be 97.4% concordant with gold standard CNV genotypes, with a missing rate of 14.7% (Table 3). These CNV genotypes had stronger association with case-control status (*P*-value of 5*e*−6) than FPC, demonstrating the value of using these genotypes to refine the association signal.

**Table 3.** CNV genotyping results at LCE3B_LCE3C locus

# GPCS	CNV genotype		
	*P*-value	Accuracy (%)	Missing rate (%)
0	1.4*e*−4	95.8	15.7
5	1.1*e*−5	97.1	14.4
20	2.7*e*−6	97.7	13.3
40	5.0*e*−6	97.4	14.7
50	1.7*e*−5	95.8	14.3

CNV genotyping accuracy, missing rates and association with psoriasis status after correction for GPCs.

## 4 DISCUSSION

While approaches for finding common SNP association with disease have become routine, finding common CNV associated with complex disease has proven more elusive. Accurately genotyping CNVs in large case–control cohorts using data from SNP genotyping arrays remains very challenging, and attempts to associate CNV genotypes defined in this way with disease have often led to highly inflated test statistics, as well as results that are difficult to independently replicate. Array CGH was used to discover the *LCE3B_LCE3C* association, and studies using array CGH tend to be more robust. However, aCGH studies are still subject to substantial batch effects, and aCGH has not been widely applied to very large multi-centre case–control cohorts needed to detect small to modest effect sizes. Now that exome sequencing is being routinely applied to case–control cohorts, there is a new opportunity to look for common CNV associations with disease. This article provides the first ‘proof-of-principle’ that exome sequencing can be used in this way, by developing an algorithm which adequately controls for batch effects, and applying this algorithm to re-discover one of the few confirmed common CNV associations with complex disease.

We have also shown that as well as detecting CNV association with disease, it is possible to calculate highly accurate absolute copy number genotypes at associated loci. To do this, we have applied cnvHap to perform joint CNV segmentation and genotyping. cnvHap has been shown to be one of the most sensitive CNV discovery and genotyping algorithms, due to its flexible modelling of intensity data (in this case read-depth data) across an entire population.

Application of our method yielded an association *P*-value between *LCE3B_LCE3C_del* and psoriasis of 7.2*e*−5 using FPC corrected for 40 GPCs, and 5*e*−6 using estimated absolute CNV genotypes. While these *P*-values are not as significant as the initial reported *P*-value of 1.38*e*−8 (de Cid *et al.*, 2009), this initial study was based on 2831 samples while the data presented here are derived from 1500 samples. A UK replication study with 1962 samples obtained an association *P*-value of 0.03, while a larger German replication study with 2278 samples obtained an association *P*-value of 3.3*e*−5 (Riveira-Munoz *et al.*, 2011).

Principal components are widely used to adjust for the inflation of test statistics due to population structure in genome-wide association studies; however, these studies typically only adjust for up to 10 PCs, with a greater number of PCs required for highly structured populations. The fact that 40 PCs were required to correct for read-depth biases likely reflects the fact that the ‘batch’ size consisted of two samples per capture array.

We might speculate that CNV of genes, and more specifically exons, may generate substantial phenotypic variation. Our ability to assess this effect to date has been limited by the technical challenge of array technologies. Exome sequencing, however, as it is measuring precisely this type of variation in the genome, provides an exciting opportunity to finally assess the association between gene copy number and phenotype.
